# The views of key leaders in South Africa on implementation of family medicine: *critical role in the district health system*

**DOI:** 10.1186/1471-2296-15-125

**Published:** 2014-06-25

**Authors:** Shabir Moosa, Bob Mash, Anselme Derese, Wim Peersman

**Affiliations:** 1 Department of Family Medicine, Faculty of Health Sciences, University of Witwatersrand, Johannesburg, South Africa; 2 Division of Family Medicine and Primary Care, Stellenbosch University, Stellenbosch, South Africa; 3 Department of Family Medicine, Ghent University, Ghent, Belgium

**Keywords:** Family medicine, Primary health care, South Africa, Health systems, Family physician, Key opinion leaders

## Abstract

**Background:**

Integrated team-based primary care is an international imperative. This is required more so in Africa, where fragmented verticalised care dominates. South Africa is trying to address this with health reforms, including Primary Health Care Re-engineering. Family physicians are already contributing to primary care despite family medicine being only fully registered as a full specialty in South Africa in 2008. However the views of leaders on family medicine and the role of family physicians is not clear, especially with recent health reforms. The aim of this study was to understand the views of key government and academic leaders in South Africa on family medicine, roles of family physicians and human resource issues.

**Methods:**

This was a qualitative study with academic and government leaders across South Africa. In-depth interviews were conducted with sixteen purposively selected leaders using an interview guide. Thematic content analysis was based on the framework method.

**Results:**

Whilst family physicians were seen as critical to the district health system there was ambivalence on their leadership role and ‘specialist’ status. National health reforms were creating both threats and opportunities for family medicine. Three key roles for family physicians emerged: supporting referrals; clinical governance/quality improvement; and providing support to community-oriented care. Respondents’ urged family physicians to consolidate the development and training of family physicians, and shape human resource policy to include family physicians.

**Conclusions:**

Family physicians were seen as critical to the district health system in South Africa despite difficulties around their precise role. Whilst their role was dominated by filling gaps at district hospitals to reduce referrals it extended to clinical governance and developing community-oriented primary care - a tall order, requiring strong teamwork. Innovative team-based service delivery is possible despite human resource challenges, but requires family physicians to proactively develop team-based models of care, reform education and advocate for clearer policy, based on the views of these respondents.

## Background

Reform of primary care service delivery towards integrated, person-centred and multidisciplinary team-based care is an international imperative [1,2]. However there is little evidence of this in African primary health care (PHC) and human resource (HR) policy [3,4]. African family physicians have felt the need to clearly define family medicine in Africa [5], but there are still difficulties in making sense of generalism in South Africa (SA) [6]. Although there has been specific policy on family medicine in countries such as Kenya [7], there has been no such clarity in SA.

Most of the generalist doctors in SA, including the majority in private general practice, enter practice, as registered medical practitioners, after undergraduate training alone. In the public service generalist doctors work in regional and district hospitals. Their limited presence in some community health centres and clinics in the district health service is as a referral support to nurses (who dominate the provision of first contact care). Most private general practitioners (GPs) work in solo practices with some in partnerships or medical corporates servicing those sections of the population with voluntary private medical insurance or cash.

Postgraduate training in family medicine has been happening on a part-time distance learning basis in SA since the 1970s, mostly in the private sector [8]. Family medicine training has achieved post-apartheid respectability by responding to public service needs, especially with surgical, anaesthetic and procedural skills at rural district hospitals. They have also been supportive in mentoring or teaching nurses who perform the bulk of primary care in the public service. The speciality has only been fully registered in South Africa since 2007 [9]. Family physicians, trained in full-time trainee specialist posts, only started entering the health system as family physicians in 2011.

There are important policy initiatives underway in South Africa. The introduction of National Health Insurance (NHI) in 2011 was intended to secure universal health coverage by 2025. Re-engineering of PHC, as a priority for the NHI, has three streams: 1) district clinical specialist teams (DCSTs) (including a single obstetrician, paediatrician, family physician, midwife, paediatric nurse and PHC nurse per district mostly to address clinical governance and training around maternal and child health outcomes); 2) school health teams; and 3) municipal ward-based PHC outreach teams (as a community-oriented primary care approach borrowed from Brazil but with no role in South Africa for family physicians) [10]. Whilst family physicians are part of the DCST they appear marginally addressed in the broader district health service and HR policy. HR policy focuses on nurses and other specialists [11].

A previous Africa-wide study on the implementation of family medicine included four respondents from South Africa [12,13]. South Africa has progressed considerably in recognising family medicine and training family physicians, however there is no literature on the views of any stakeholders on family medicine. The aim of this study was to explore and to understand the views of a group of governmental and academic leaders in South Africa on the contribution of family medicine to the health system, and the challenges posed to implementation and human resource policy.

## Methods

### Study design

This was a qualitative study by means of in-depth interviews of leaders in South Africa.

### Setting

The setting was the public health system (including academic centres), represented by the national department of health, nine provincial departments of health, and the eight medical schools in South Africa.

### Sampling and selection

The study population were senior leaders, perceived as influential to PHC, in this setting. Purposive sampling started with deputy-director generals in the national department of health and deans at medical schools, with snowball sampling thereafter. Geographical spread and a balance between academic and governmental respondents guided the sampling. Those with postgraduate education in family medicine were excluded. The interviews were stopped after data saturation, with repetition of themes, as identified by the first two authors (SM, RM).

### Data collection

Written informed consent was obtained before an interview lasting up to 60 minutes, which was conducted in the respondent’s office. The first two authors (SM, RM), who were trained in qualitative interviewing, conducted the interviews in English and recorded them digitally. An interview guide (Table 1) was used to help explore the viewpoint of respondents.

**Table 1:** Interview guide

1	Can you tell us about Family Medicine?
2	What are your thoughts on the role of Family Medicine in South Africa?
3	What do you think are the issues in implementing the discipline of Family Medicine?
4	What are the critical human resource issues that influence the establishment of Family Medicine?
5	Do you have anything else to add?

A research assistant transcribed the recordings verbatim. The first author (SM) validated the transcriptions against the recordings.

### Data analysis

Qualitative data analysis followed the framework method [14]. All authors familiarised themselves with the data. The first two authors’ (SM, BM) identified themes from all transcripts and developed a thematic index. All transcripts were then systematically coded according to the thematic index by the first author (SM) using NVivo 9 (QSR International, http://www.qsrinternational.com). These index-coded transcripts and draft results were then presented to all authors. The overall process was reviewed against the COREQ Checklist [15].

### Ethical approval and funding

The University of the Witwatersrand’s Human Research Ethics Committee (Medical) provided ethical approval in January 2011 (M110104). There was no monetary reward for participation. The data remains confidential, and will be destroyed after 5 years. Respondents were anonymised in all transcripts and analyses. This study was undertaken as part of the HURAPRIM Project, which received funding from the European Union’s Seventh Framework Programme (FP7-AFRICA-2010) under grant agreement no. 265727.

## Results

A total of 16 interviews were conducted with accessible leaders nationally and in five provinces (Table 2) between June 2011 and December 2012. The respondents were at the level of head of department, deputy head of department, chief director or senior consultant in the national or provincial departments of health and deans, vice-deans, heads or senior academic of medical schools. Respondents’ voices are labelled as coming from a national or provincial level, by interview number and as governmental or academic

**Table 2:** Number of respondents by sector, location and professional background

	**Government**	**Academic**	**Professional Background**
**National**	4	0	medical practitioner, psychologist, nurse x 2
**Provinces:**			
- Gauteng	1	4	manager, microbiologist, psychiatrist, public medicine specialist x 2
- Western Cape	1	1	paediatrician, medical practitioner,
- KwaZulu Natal	1	1	medical practitioner, internal physician
- Free State	1	1	medical practitioner, gynaecologist
- North West	1	0	medical practitioner with MPH
**Total**	**9**	**7**	

The major themes identified were: issues regarding the inclusion of family physicians in the health system; the roles of family physicians; and human resource issues. There were no differences between academic and government leaders’ views.

### Family physicians in the health system

#### Critical to the district health system but ambivalence about leadership role and ‘specialist’ status

Family physicians were seen as critical to improving the quality of care in district health services through clinical leadership as well as by modelling a more comprehensive and holistic style of care to both individuals and families. However there was ambivalence about who should ‘drive’ primary care. On the one hand it was felt that PHC must be ‘nurse-driven’ due to shortages of doctors and inappropriate training. On the other hand many felt that the primary care system was ineffective with patients bypassing inadequately capacitated nurses at clinics to go to hospitals and that the system should be more doctor led:

“They (family physicians) are there as the overall head of clinical services” [SA16 Gauteng Government]

“I think the concerns I have would be not to turn our primary health care to be a doctor driven primary health care” [SA09 National Government]

“I think the big problem remains the quality of clinical care that people get at clinics and community health centers, which is why they vote with their feet” [SA10 Gauteng Academic]

“Whilst one doesn’t want to be chauvinistic in terms of doctors and their needs, I think communities at this point want to see doctors leading the health care teams within the services, within the district services” [SA08 Gauteng Academic]

Whilst there was a strong belief that family physicians should be responsible for clinical leadership in the district and represent doctors on the district management team there was significant ambivalence about their management role. There were concerns that administrative functions could overwhelm their clinical role:

It was also felt that the discipline of family medicine was poorly understood and largely unrecognised within communities, national government and academic circles. The discipline was sometimes equated with private general practice and had limited status within the specialist hierarchy. Confusion in understanding the family physician appears heightened by the incongruence between their training as expert generalists and their registration as ‘specialists’:

“It's also to provide a non-specialist specialist” [SA14 KwaZulu Government]

“There is not necessarily in my mind a clear understanding across the sector from the policy makers to the executive managers, of the unique value add of the specialist in family medicine as distinguished from a generalist” [SA06 Gauteng Academic]

“I know there’s not always a clear divide between administrative responsibilities and clinical governance especially in our health settings” [SA12 National Government]

#### National health policy creates both threats and opportunities for family medicine

Respondents shared considerable disquiet about the threat of the new District Clinical Specialist Teams (DCSTs) to the district health system. They felt that DCSTs: lacked unclear accountabilities to district managers, would struggle with conflicted roles in district hospitals and was being implemented without evidence of the value of specialists in the district health system. They felt that family physicians were best suited for the district health system. Some felt that conflicts between specialists and family physicians were normal, with overlapping roles, and that family physicians should define their roles more confidently:

“Very complicated. You see the more people you put into a system, the more complex it becomes… we experimenting with this thing” [SA12 National Government]

“The generalist actually has to call the shots and tell the specialist where they come in” [SA05 WesternCape Academic]

National Health Insurance (NHI), especially Primary Health Care (PHC) Re-engineering, was seen as an opportunity for family physicians to lead the health care teams in community health centres for defined populations:

“I think with NHI there’s a huge opportunity for Family Medicine [SA02 National Government]

“Under any of the models of the NHI that might be attained, it is my view that the family medicine doctor should, ideally, be the leader of the health care team in the health center. [SA06 Gauteng Academic]

### Roles of family physicians

#### Providing expert generalist care to referred patients and reducing referrals to secondary or tertiary hospitals

Family physicians were seen as critical in terms of managing patients referred from junior clinicians or nurses and reducing referrals to secondary or tertiary hospitals. When referrals were necessary they had more authority to liaise with other specialists. There were concerns with the lack of family physician posts at district hospitals, poor referral systems and the breadth of clinical competency required by family physicians:

“They (family physicians) would look at providing services referred from clinics…. Any problematic stuff that can't be dealt with gets sent to the CHC (Community Health Centres). ….So, I would say put them in the CHC's” [SA11 National Government]

“It also reduces the necessary referrals to the level two hospitals….. allow(ing) doctors (there) to take referrals in a proportion that is very well managed..… family medicine can play that role: to isolate, to clean up clinically” [SA09 National Government]

#### Taking responsibility for clinical governance and improving the quality of care

There was a widespread concern regarding the poor quality of care at clinics, health centres and district hospitals. Leaders felt that family physicians could make a significant contribution to this by taking responsibility for clinical governance, quality improvement and training other team members such as nurses, junior doctors or general practitioners:

“One of the benefits for me is to start at clinical governance to help with the transfer of skills to nurses, so that they can start working side by side with doctors” [SA09 National Government]

#### Providing support to more community-oriented care

Family physicians were expected to understand public health and develop a community-oriented approach towards defined populations. Family physicians could also support the development of family practices, even with nurses looking after their own family practices, which would provide more continuity of care for defined groups of patients:

“Family medicine for me has a major role to play….especially in terms of supporting services of the clinics and at a household level…you need somebody with a much wider understanding of public health” [SA11 National Government]

“I believe that we need to reorganize and re-engineer the delivery of primary health care in health centers and clinics in the country around the model of ….discernible family practices” [SA06 Gauteng Academic]

### Human resource issues

#### Consolidating the development and training of family physicians

There was confidence in the new full-time training, with rotations through various specialties. However there was disquiet about the quality of graduates from the old part-time postgraduate training. It was felt that training in management, health information, and community-based epidemiological research would be useful:

“The new curriculum I think it has a lot of promise” [SA12 National Government]

Respondents felt that South Africa needed to drastically increase the number of family physicians. It was felt that postgraduate training needs to be increased, and suggested attracting South Africans trained as doctors in Cuba and training private GPs in their practices:

“If you know the family medicine physicians can pick themselves up and create enough specialists” [SA07 Gauteng Academic]

Family physicians were expected to be uniformly trained, albeit with flexibility for different contexts. However it was felt that the clinical skills required, particularly for district hospitals and remote locations, were too extensive. A minor view advocated for ‘sub-specialisation’, with better remuneration:

“If you insist in having a (academic) department of family medicine or something I must have a clear sub-department rural medicine, a clear sub-department of general practice and a clear sub-department of I don’t know what we are going to call it, ‘non-general practice primary care’ you know. Hospital based primary care or something. To me they are three very separate things… ….I would almost plea for a bit of specialization” [SA13 KwaZulu Natal Academic]

Respondents felt that undergraduate training was dominated by specialists and needed family physicians to take the lead in re-orientating it to service delivery and a team approach in community-based education and research.

#### Shaping human resource policy to plan for family medicine

The poor performance of the health system was seen as an important challenge. Whilst some of it was due to the legacy of apartheid it was also felt that corruption and poor management were contributing factors:

“(The) South African health service is in a mess. It is poorly managed” [SA03 Western Cape Government]

They felt that the serious shortage of doctors and family physicians in primary care - both in number and distribution - favoured a nurse-based approach. Provinces with more family physicians were able to define roles better. Planning needed to be premised on a team approach that included family physicians throughout the district:

“(Family medicine) is not going to work unless there is visible evidence that there’s a proper caring structure at the district level…. this is a team approach” [SA08 Gauteng Academic]

“Just to say that ideally you would like to have a family physician in every primary health care setting, in every district” [SA11 National Government]

Family physicians were called on to develop norms for primary care in terms of a service package for a defined population and models for implementation of NHI. They suggested a task-based planning approach with the available skills mix of the primary health care team, including family physicians. Respondents were very open to human resource plans from family medicine but stressed that this planning needed to be arranged to account for various roles, rural challenges, evidence and progression over time. It was felt that oversimplifying the discipline of family medicine would be a threat:

“What would be very useful is if the family physicians took the primary health care package and said in terms of the delivery of these services to the population what would our role be?” [SA11 National Government]

“You can arrange your inputs and your processes, however you want to, with the evidence that exists” [SA12 National Government]

“Now in time as we produce lots more doctors and lots more family medicine specialists, this model may refine over time where the family medicine specialist may become one, two, three or four themselves, at the heart of that. And start to care for a community in that area” [SA06 Gauteng Academic]

Other issues of human resource management were also mentioned. Despite respondents’ feeling that few young doctors were attracted to family medicine as a career path, they felt that the specialist level remuneration and supervisory support from senior family physicians could change this. Planning also needed to address management of change. They felt that it would require family physicians, as a group, to plan and position themselves within small demonstrable sites and develop evidence of impact. Family physicians needed to actively advocate for their specialty based on clearer roles, specific models and contributions based on the principles and uniqueness of family medicine, and evidence:

“It’s about fighting for that space and how do you fight for space? By doing what you have got to do and making an impact” [SA09 National Government]

“This I can’t do but this I can do, this is the contribution I can make…..you need to market it but not only by social strata placement but by what we do’ [SA09 National Government]

## Discussion

The results resonate well with the aim of this study: to understand the views of governmental and academic leaders in South Africa on the contribution of family medicine to the health system, and the challenges posed to implementation and human resource policy.

Whilst family physicians were seen as critical to the district health system there was ambivalence on their leadership role in the district health system and their ‘specialist’ status in the realm of generalism. National health policy was creating both threats and opportunities for family medicine. Whilst there was ambivalence and caution about teamwork, given existing human resource shortages, leaders’ views in South Africa were consistent with the findings of the previous study in Africa that family physicians are critical to the district health system (comprising clinics, community health centres (CHCs) and district hospitals). There was also consistency in lacking a strong positive understanding of generalists [13]. This is evident in respondents’ hospital and ‘departmental’ bias with family physicians being directed towards staffing district hospitals due to human resource shortages. This is a major paradigmatic challenge in South Africa, and Africa, where reductionist specialism dominates the discourse [6]. Family physicians in South Africa need to define and advocate generalism and family medicine as a set of values and principles in primary care used by the whole team, including family physicians.

Three key roles for family physicians emerged: providing expert generalist care to patients referred from nurses and junior doctors at clinics and community health centres, and thus reducing referrals to secondary or tertiary hospitals; taking responsibility for clinical governance and improving the quality of care; and providing support to community-oriented care. Roles were strongly defined around district hospital care, but did include an awareness of the need to shift towards community-orientated care, more so than African respondents [13] but consistent with African family physicians’ desires [16,17]. Respondents saw the presence of family physicians as an opportunity to rearrange the health system, with National Health Insurance (NHI), to achieve both equity *and* quality. Higher expectations in terms of quality may not be far off with growing populations and urbanisation projected to make South Africa part of the largest middle class globally by 2060 [18].

Human resources appear to be the major challenge in implementation. Whilst respondents felt that developing a career path for generalist doctors was an important issue they seemed not to recognise the problems of working conditions that contributes strongly to the emigration of doctors [19]. Respondents’ spoke of consolidating the development and training of family physicians, and shaping human resource policy to include family medicine. Respondents supported the African model of decentralized, scaled-up training (including private GPs) and undergraduate reform, consistent with African leaders [13]. There were valid questions as to the skills required for family physicians in the NHI, especially team-based epidemiological management of populations. Whilst the idea of sub-specialisation might go against the concept of generalism it might also enable the wide skills range expected of family physicians in Africa (from community-oriented primary care to inpatient surgical/anaesthetic skills) to be better structured during training and better remunerated within governments occupation-specific dispensation (OSD) human resource system. As more family physicians are trained in a shorter time to work more widely as expert generalists in team-based ambulatory care (including a community health centre) in the NHI environment there are skills sets e.g. hospitalist care, rural health, management or education that could be deferred to longer elective training as ‘sub-specialties’ or special interests. This may be part of long term planning and may allow family physicians to serve a community more comprehensively without trying to be ‘all things to all people’. Revised models of training may be required, including undergraduate medical doctors and the range of members of the PHC team, to scale up for expected impact in South Africa [20].

Innovative solutions (e.g. including the large number of general practitioners (GPs) from the current private sector into a strongly regulated capitated public primary care system) can reduce the shortage of doctors in the public service [21] but team-based primary health care will still remain an important consideration. Family physicians need to be proactive in defining primary care service packages for primary care, staffing mixes and skills, including the range of members of the Primary Health Care (PHC) team. Family physicians also need to redefine the skills they require with a team-based approach, addressing the primary health care needs of a defined population for first contact care that is comprehensive, coordinated, continuous and accountable. These South African government and academic leaders recommended reviewing structure and roles within the primary health care team using business process re-engineering ideas. The World Health Organisation’s suggested tool for developing staffing norms, Workload Indicators for Staffing Needs (WISN), can be useful [22].

The strengths of the study were the wide geographic and professional variety of respondents, the strong adherence to the interview guide and the communication skills of two well-trained interviewers. The findings provide useful and transferable insights to the development of family medicine. A limitation may be that leaders more easily accessed by family physician researchers produced a more favourable view of family medicine.

## Conclusions

Stakeholders in primary care should be actively engaged by family physicians to develop revised PHC service packages, PHC team models and evidence-based human resource policy proposals for integrated PHC teamwork (inclusive of family physicians) under universal health coverage [23].

## Competing interests

The authors declare that they have no competing financial or non-financial interests.

## Authors’ contributions

SM and BM conceived and designed the study, drafted the protocol and did the interviewing. SM and BM developed the thematic index. SM coded the data and presented the themes and findings. The authors (SM, BM, AD and WP) collectively interpreted this by email and drafted the manuscript. All authors (SM, BM, AD and WP) read and approved the final manuscript.

## Authors’ information

SM: MBChB, MFamMed, MBA, is a senior clinical lecturer in the Department of Family Medicine and the lead researcher in the HURAPRIM Project at the University of the Witwatersrand, South Africa.

BM: MBChB, MRCGP, DRCOG, DCH, FCFP, PhD, is Head of the Division of Family Medicine in Stellenbosch University, South Africa.

AM: MD, PhD, is a Professor in the Department of Family Medicine, Ghent University, Belgium.

WP: MSc, PhD is a Researcher and Scientific Coordinator of the HURAPRIM Project in the Department of Family Medicine, Ghent University, Belgium.

## Pre-publication history

The pre-publication history for this paper can be accessed here:

http://www.biomedcentral.com/1471-2296/15/125/prepub

## References

[B1] World Health Organization The World Health Report 2008: Primary Health Care – Now More than Ever 2008 Geneva

[B2] Sixty-second World Health Assembly Primary health care, including health system strengthening World Health Assembly 2009 1 3

[B3] World Health Organisation Report on the Review of Primary Health Care in the African Region 2008 Mauritius

[B4] WHO Regional Committee for Africa Road Map for Scaling up the Human Resources for Health for Improved Health Service Delivery in the African Region 2012–2025 2012 Luanda

[B5] Mash RB Reid S Statement of consensus on family medicine in Africa African J Prim Heal Care Fam Med 2010 2 4

[B6] Howe A Mash R Hugo J Developing generalism in the South African context S Afr Med J 2013 103 899 900 10.7196/samj.7509 24300624

[B7] Ministry of Health Kenyan Family Medicine Strategy 2007 Nairobi

[B8] Levenstein JH The South African general practitioner - an overview S Afr Med J 1981 59 309 310 7466536

[B9] Health Professions Council of South Africa Regulations Relating To The Registration Of Specialities And Subspecialities In Medicine And Dentistry: Amendment. Volume No. R. 712 2007 Pretoria: Republic of South Africa: Republic of South Africa

[B10] National Department of Health Provincial Guidelines for the Implementation of the Three Streams of PHC Re-Engineering 2011 Pretoria 1 10

[B11] National Department of Health Human Resources for Health South Africa: HRH Strategy for the Health Sector: 2012/13 – 2016/17 2011 Pretoria

[B12] Moosa S Downing R Essuman A Pentz S Reid S Mash R African leaders’ views on critical human resource issues for the implementation of family medicine in Africa Hum Resour Health 2014 12 2 10.1186/1478-4491-12-2 24438344 PMC3899624

[B13] Moosa S Downing R Mash B Reid S Pentz S Essuman A Understanding of family medicine in Africa: a qualitative study of leaders’ views Br J Gen Pract 2013 63 209 216 10.3399/bjgp13X664261 PMC3582980 23561788

[B14] Ritchie JSL Bryman A, Burgess R Qualitative data analysis for applied policy research Anal Qual Data 1993 London: Routledge 173 194

[B15] Tong A Sainsbury P Craig J Consolidated criteria for reporting qualitative research (COREQ): a 32-item checklist for interviews and focus groups Int J Qual Heal care J Int Soc Qual Heal Care/ISQua 2007 19 349 357 10.1093/intqhc/mzm042 17872937

[B16] Mash B Downing R Moosa S De Maeseneer J Exploring the key principles of Family Medicine in sub-Saharan Africa: International Delphi consensus process S Afr Fam Pract 2008 50 60 65

[B17] Kark S The Practice of Community-Oriented Primary Health Care 1981 New York: Appleton

[B18] African Development Bank Africa in 50 Years’ Time 2011 Tunis

[B19] Moosa S Wojczewski S Hoffman K Poppe A Nkomazana O Peersman W Willcox M Maier M Derese A Mant D Why there is an inverse primary-care law in Africa Lancet Glob Heal 2013 1 e332 e333 10.1016/S2214-109X(13)70119-0 25104594

[B20] De Maeseneer J Scaling up family medicine and primary health care in Africa: statement of the primafamed network, Victoria Falls, Zimbabwe African J Prim Heal Care Fam Med 2013 5 5 7

[B21] Moosa S Luiz J Carmichael T Introducing a national health insurance system in South Africa: a general practitioner’s bottom-up approach to costing S Afr Med J 2012 102 794 797 23034207 10.7196/samj.6072

[B22] Shipp PJ Workload Indicators of Staffing Need (WISN): A Manual for Implementation 1998 Geneva: WHO Division of Human Resources Development and Capacity Building

[B23] Hernan Montenegro - Which approach for high quality primary care? [http://www.youtube.com/watch?v?=?vjn2b71k5ho]

